# The mental health of the UK Armed Forces: where facts meet fiction

**DOI:** 10.3402/ejpt.v5.23617

**Published:** 2014-08-14

**Authors:** Elizabeth J. F. Hunt, Simon Wessely, Norman Jones, Roberto J. Rona, Neil Greenberg

**Affiliations:** 1 Academic Centre for Defence Mental Health (ACDMH), King's College London, Western Education Centre, London, UK; 2 King's Centre for Military Health Research (KCMHR), King's College London, Western Education Centre, London, UK

**Keywords:** Military, combat, service personnel, veterans, reservists, post-traumatic stress disorder, delayed-onset PTSD, mild traumatic brain injury, Trauma Risk Management

## Abstract

A substantial amount of research has been conducted into the mental health of the UK military in recent years. This article summarises the results of the various studies and offers possible explanations for differences in findings between the UK and other allied nations. Post-traumatic stress disorder (PTSD) rates are perhaps surprisingly low amongst British forces, with prevalence rates of around 4% in personnel who have deployed, rising to 6% in combat troops, despite the high tempo of operations in recent years. The rates in personnel currently on operations are consistently lower than these. Explanations for the lower PTSD prevalence in British troops include variations in combat exposures, demographic differences, higher leader to enlisted soldier ratios, shorter operational tour lengths and differences in access to long-term health care between countries. Delayed-onset PTSD was recently found to be more common than previously supposed, accounting for nearly half of all PTSD cases; however, many of these had sub-syndromal PTSD predating the onset of the full disorder. Rates of common mental health disorders in UK troops are similar or higher to those of the general population, and overall operational deployments are not associated with an increase in mental health problems in UK regular forces. However, there does appear to be a correlation between both deployment and increased alcohol misuse and post-deployment violence in combat troops. Unlike for regular forces, there is an overall association between deployment and mental health problems in Reservists. There have been growing concerns regarding mild traumatic brain injury, though this appears to be low in British troops with an overall prevalence of 4.4% in comparison with 15% in the US military. The current strategies for detection and treatment of mental health problems in British forces are also described. The stance of the UK military is that psychological welfare of troops is primarily a chain of command responsibility, aided by medical advice when necessary, and to this end uses third location decompression, stress briefings, and Trauma Risk Management approaches. Outpatient treatment is provided by Field Mental Health Teams and military Departments of Community Mental Health, whilst inpatient care is given in specific NHS hospitals.

The current interest in comparing and attempting to explain differences between different nations military health is by no means a new phenomenon. Great Britain and the United States have a history of military research collaboration dating back to the First World War when the American National Research Council and British Medical Research Committee jointly published a medical bulletin focusing on the health problems of war (Morley Fletcher, 1919). This research alliance was strengthened during the Second World War (Casper, 2008), though became fraught when American physicians accused the British of “minimizing the problem” of civilian neuroses in the British population caused by air raids. Jones writes that “the American allegation not only presented the possibility that civilians were not coping as well with the air raids as the triumphant newspaper headlines suggested, it also trivialized the British medical community's response and painted it as either intentionally evasive or emotionally repressed” (Jones, Woolven, Durodie, & Wessely, 2004).

Little research into the mental health of serving members or veterans of the UK Armed Forces was undertaken between 1945 and 1995. However, the emergence of the so called “Gulf War Syndrome” amongst personnel who took part in the 1991 Gulf War was a stimulus for change, and led to the commissioning of population-based research into the health and well-being of UK Gulf War veterans. A second stimulus was the large class action brought in 2002/3 by veterans claiming that the Ministry of Defence (MoD) had failed to address the issue of post-traumatic stress disorder (PTSD) (McGeorge, Hacker-Hughes, & Wessely, 2006). The class action failed, but the combination of this and memories of the Gulf War Syndrome saga encouraged the planning of a new cohort study into the physical and mental health of military personnel taking part in the 2003 invasion of Iraq. This study had to be extended with increased UK operations in Afghanistan, and remains ongoing, providing the core of the data for this review. This has also had the benefit of enabling the sharing of military health experiences and research between the UK and other nations, particularly the US, Canada, and Australia.

In recent years the UK has been working closely with the US, and is even currently undertaking specific research projects funded by the US Department of Defence, such as studying the effectiveness of post-operational screening for mental health problems. Furthermore, many of the issues of military mental health have been explored by US and UK researchers, using similar tools on service personnel deployed to the same conflicts. This context has allowed identification of areas of both similarity and contrast, which has informed debate into differences between countries, settings and perspectives.

This article will describe the research efforts of the last 12 years on the mental health of UK military personnel. The main outcomes in most of the studies are PTSD, common mental disorders, alcohol misuse, multiple physical symptoms and, more recently, mild traumatic brain injury (mTBI) and violence. Most of the issues will be related to the Iraq and Afghanistan deployments, but will touch upon Gulf War deployment too. The issues that will be discussed in this review are the prevalence of mental ill health and associated events, the increase in deployment tempo (specifically the possible consequences of overstretch on mental illness), the mental health impact of deployment in reserve personnel, and the research into the development of support services to tackle mental health issues.

## Post-traumatic stress disorder

Given that PTSD only became a discrete diagnostic category in 1980 (American Psychiatry Association, 1980) and that UK research efforts to examine the disorder in members of the UK Armed Forces were initially sluggish, there was a paucity of data until the start of the new millennium.

A modestly sized retrospective study of UK Armed Forces peacekeepers deployed between 1991 and 2000 yielded a PTSD prevalence rate of 3.6% when a PTSD Checklist-Military (PCL-M) score of 50 was used (Greenberg, Iversen, Hull, Bland, & Wessely, 2008). The PCL-M is a 17-item self-report measure of the DSM-IV symptoms of PTSD, which asks about symptoms in response to stressful military experiences; there is also a similar civilian version (PCL-C). These rates were lower than those reported in a study of US peacekeepers deployed to Somalia where a PTSD rate of 8% was found (Litz, Orsillo, Friedman, Ehlich, & Batres, 1997), even though in the US study the PCL cut-off score was stricter at 68. Given the potentially very different exposures experienced during the two deployments, a direct comparison of the rates, even factoring in the stricter PCL score in the US peacekeeper study, may still be misleading.

A study carried out from 2001 to 2002 (prior therefore to the start of the Iraq War) of a representative sample of the UK Armed Forces found a PTSD prevalence of 2.5% (Rona, Jones, French, Hooper, & Wessely, 2004). Since then, the core of the UK research effort has been the study of a large, representative sample of UK Armed Forces personnel initially sampled at the time of the 2003 Iraq War (Hotopf et al., 2006), which has been undertaken by a team of independent researchers at King's College London, members of the King's Centre for the Military Health Research (KCMHR). The first stage of this study concluded in 2005. At that time PTSD rates were 4.4% in the sample of those who had been deployed to Iraq (“deployed” sample). This rate was not statistically different to the rate of 3.5% found in those who had not been deployed to Iraq (“non-deployed” sample). It should be noted that personnel in this “non-deployed” sample may have been on other operational deployments during their military careers. The small non-significant difference between deployed and non-deployed could be explained by the higher PTSD rate of 5.7% amongst combat troops. This fairly low PTSD prevalence in deployed personnel was found despite their high combat exposure, with the majority reporting at some point thinking they might be killed, or coming under mortar, SCUD missile, or artillery fire. The PTSD rate in a similar US study was higher (12.6%), despite using an identical measure and case definition (Hoge et al., 2004). We will consider possible explanations for these differences later. Rates of PTSD were also higher in a study of Australian troops, with a 12-month prevalence of 8.3% (Mental Health in the Australian Defence Force, 2010). Rates in the German military were much lower at 2.9% according to recent research (Schulte-Herbruggen, & Heinz, 2012; Wittchen et al., 2012). They were lower again in Canadian troops at 2.3% (Sareen et al., 2007). However, it is important to note that these Australian, German and Canadian studies used a very different measure than UK and US studies; the former three used the Composite International Diagnostic Interview (CIDI), which measures the 12-month prevalence rate of PTSD (rather than the 1-month prevalence of the PCL) and is a face-to-face interview rather than questionnaire. Furthermore, the versions of the CIDI used also varied between these countries; Canada used version 2.1, whilst Australia used version 3, which has a significantly higher rate of indexed traumas and asks both about the worst and a random event; Germany used the computer-assisted Munich CIDI military version.

The mental health of United Kingdom Armed Forces (UKAF) personnel has also been assessed during, as opposed to before or after, deployment, using Operational Mental Health Needs Evaluations (OMHNEs). These were undertaken in Iraq in 2009 (Mulligan et al., 2010), and Afghanistan in 2010 (Jones, in press-a) and 2011 (Jones, in press-b) and are broadly similar to the US MHAT studies, on which they were modelled. These have found a decreasing trend in the rate of PTSD for the period; in 2009 the prevalence was 3.4%, in 2010 it was 2.7%, and 1.9% in 2011. A sub-group analysis of the 2010 Afghanistan data found that personnel in Forward Operating Bases (FOBs) and Patrol Bases (PBs) were twice as likely to suffer from probable PTSD (4.2%) as personnel in the Main Operating Bases (MOBs) (1.9%) (Jones, in press-a).

Whilst the majority of UK and US studies of recent years have used PCL scores of 50 or more to estimate rates of probable PTSD, both UK and US studies have used PCL scores of 30 or more to provide an estimate of symptom burden (though not of a clinical disorder), with empirical evidence demonstrating a significant level of functional impairment in those with a PCL of 30 or over (Rona, Jones, Iversen, & Hull, 2009). Using this a priori lower cut-off for PCL cases, what emerged was a clear trend for increasing PCL cases with increasingly forward, and hence more dangerous, deployment. The rates in Afghanistan in 2010 were 15.5% in the MOBs, 20.6% for FOBs, and 29.1% for PBs (Jones, in press-a). A similar trend was found in 2011 (Jones, in press-b), with substantial PTS symptoms in 10.3% of personnel in MOBs, 11.1% in FOBs, 16.7% in PBs and 17.4% in Check Points (CPs). These findings further illustrate the correlation of combat exposure with higher PCL scores. The rates were highest (20.6%) in partnering teams, where personnel train or work closely with the Afghan National Army and Police.

The OHMNE studies have also demonstrated an apparent protective effect of perceived good cohesion and leadership; well-led and close-knit units had substantially better mental health, even if they had been exposed to high-threat situations. For example, the Afghanistan 2010 OMHNE found that the impact of experiencing high levels of traumatic exposures upon general mental health was substantially reduced, to the point of non-significance, by good unit leadership and high morale (Jones, in press-a). Similar findings have been found in other larger studies, which also had the advantage of having analyses adjusted for possible confounders (Rona, Hooper, et al., 2009; Sundin, Fear, Hull, et al., 2010). It has been proposed that this may be because unit cohesion is associated with individual resilience and adaptive coping with traumatic experiences (Brailey, Vasterling, Proctor, Constans, & Friedman, 2007; Whealin et al., 2007). It is difficult to fully unravel the direction of cause and effect between cohesion/leadership and mental health however, since those already suffering from mental health difficulties are more likely to be self-isolative or have a negative view of events and people around them, and thus to report worse cohesion and poorer leadership.

It would be ideal if we could make direct comparisons between the mental health of the population from whom the military is recruited from. The adult psychiatry morbidity in the 2007 England survey found that, amongst the general English population living in private households, 3.0% of adults screened positive for current PTSD (McManus, Meltzer, Brugha, Bebbington, & Jenkins, 2009). Men between ages 16–24 were more likely to have current PTSD with a screening rate of 5.1%, dropping to 3.6% in the 25–34 years bracket. Whilst this suggests that the PTSD rates in the British military are similar to those of the general population, there remain significant differences between the methodologies of the 2007 England survey and the UK military questionnaire based studies, including the use of face-to-face interviews and the measurement of prevalence over the past week not the last month. It also must be remembered that the British Armed Forces are a highly heterogeneous population containing significant numbers of personnel not only from Great Britain but also from Foreign and Commonwealth nations, and thus this English survey is by no means representative of all these populations.

However, the 2007 survey of England was able to make direct comparisons between both national service veterans and their age and sex-matched veterans, and post-national service veterans and their controls (Woodhead et al., 2011a, 2011b). These found no statistical significant effect on PTSD rates of their military backgrounds. Data protection restrictions mean that at present it is not permitted for similar direct comparisons to be made for the modern generation of soldiers relevant to this review.

### Delayed-onset PTSD

A classic paper from the US by Lee et al. suggested that true delayed-onset PTSD (defined as the onset of probable PTSD at least 6 months after a traumatic event) was not common in military samples. This was a 50-year prospective study of American World Ward II veterans which found that 16 men who had high combat scores but reported no PTSD symptoms in 1946 could not recall ever having had such symptoms in 1988, whilst only three men who reported no PTSD symptoms in 1946 then reported such symptoms in 1988 (Lee, Vaillant, Torrey, & Elder, 1995).

However, there has been a growing interest in the possibility that delayed PTSD could be more common than initially thought. A US study indicated that PTSD rates were increasing with time since return from deployment in the US (Milliken, Auchterlonie, & Hoge, 2007). This was not previously thought to be the case in the UK, with a systematic review in 2007 showing that delayed-onset PTSD with lack of any previous PTSD symptoms was uncommon (Andrews, Brewin, Philpott, & Stewart, 2007). UK studies have suggested that whilst some troops experience trauma-related symptoms during the first few weeks or months after returning from deployment, a significant proportion of those that develop PTSD are often unwilling to seek help and may only do so when persuaded by others they trust (Iversen et al., 2011) thus delayed *presentation* of PTSD may occur whilst delayed-onset PTSD was thought to be rare in the UK military.

A recent paper by Goodwin et al. demonstrated the complexities related to delayed PTSD. The authors found that delayed-onset PTSD represented 46% of all PTSD cases assessed within their sample of UK military personnel (Goodwin et al., 2012). However, they also showed that 27.2% of those who had gone on to develop delayed-onset PTSD had sub-threshold PTSD symptoms at baseline (compared to just 3.6% for those who did not then develop PTSD). This also demonstrates the potential significance for the future of sub-threshold symptoms. The complex course of PTSD was emphasised by a 20-year longitudinal study in veterans of the 1982 Lebanon War, showing PTSD rates dropping 3 years post-war and rising again 17 years later (Solomon & Mikulincer, 2006). It should be borne in mind that delayed-onset PTSD is a difficult area to research as traumatic experiences may also occur outside deployment operations between the assessments.

It is important to note that even if delayed-onset PTSD is more common than previously believed, this does not represent a major overall increase in PTSD prevalence rates, since many other cases will be getting better, whilst some individuals become symptomatic. This is illustrated by a report showing that two thirds of those with PTSD at phase 1 of the study subsequently had a PCL score below 30 at phase 2 of the study (Rona, Jones, Sundin, et al., 2012). Thus, this does not seem to represent the “tidal wave” of military personnel mental health breakdown that the UK press seems to predict (http://www.telegraph.co.uk/news/uknews/defence/7716014/Medical-journal-warns-of-tidal-wave-of-mental-trauma-among-servicemen.html), but rather a similar picture to what is known about the prevalence of common mental disorders in the general population; that there is a constant interchange between improvement, relapse and recovery.

Another recent study looking at predictors of delayed-onset PTSD occurring after military discharge in those subsequently awarded war pensions found that whilst the veterans with delayed-onset PTSD were indistinguishable from controls with no PTSD on their psychiatric presentation in service according to their medical records, their personnel records revealed that they had significantly greater rates of indiscipline prior to exposure to potential trauma (Brewin, Andrews, Hejdenberg, & Stewart, 2012). In spite of the difference in the number of offences in the personnel developing PTSD after discharge, there was not a significant difference between the personnel with PTSD in service and the control group who never developed PTSD at any time. The authors argued that the various forms of early and late-developing PTSD may be different conditions with different genetic risk factors and different natural histories and should not be included together.

### Discussion on PTSD rates in UK Armed Forces

So why do rates of PTSD appear to be lower in UK than in US personnel, despite similar measures of prevalence being used (in contrast with other allied nations)? A number of explanations have been put forward (Sundin, Fear, Iversen, Rona, & Wessely, 2010). These can be grouped as follows: differences in combat exposure, demographic differences between the UK and other troops, differences in study design, policy differences and cultural perspectives.

#### Combat role and combat exposure

Some US studies have concentrated on personnel in combat roles, whom one would expect to have a greater number of threatening experiences than combat support (e.g., engineers or signallers) or combat services support (e.g., administrative or medical services) personnel, though it is important to note that there are also very high combat exposures in non-combat arms, for example Combat Medical Technicians who patrol with combat personnel in order to provide immediate aid if soldiers are wounded, or Counter-IED teams who are specially trained engineers who defuse roadside bombs. The American Millennium Cohort based on personnel from all services has found that of those US personnel who reported combat exposure, 7.3–8.3% met criteria for PTSD (using a PCL checklist score cut-off of 50) after excluding approximately 3% of the population with previous history of PTSD (LeardMann, Smith, Smith, Wells, & Ryan, 2009; Ryan et al., 2007; Smith et al., 2008), whilst PTSD rates were considerably lower (1.4–2.1%) in deployers who did not report combat exposure (Smith et al., 2008). However, it is important to distinguish between combat exposure and having a combat role, as some who do not have a combat role may still experience combat exposure.

When only UK and US combat troops are compared, the UK rates are still significantly lower. There may be a difference in the level and type of combat which different forces are engaged. For example, in the 2003 Iraq study 53% of UK personnel reported coming under artillery, rocket, or mortar attack, compared with 86–92% for the studied US forces (Hoge et al., 2004). A more recent study, of Army personnel who had deployed to Iraq in 2007–2008, which also used self-reported combat exposures, again showed that the US sample described having more combat exposures overall and were more likely to report handling human remains, being in a threatening situation where they were unable to respond, directing fire at the enemy, clearing/searching buildings and encountering sniper fire than the UK sample (Sundin et al., 2014). In contrast, the UK sample was more likely to report indirect fire.

It is difficult to compare combat exposures directly rather than by self-report; however, fatality rates seem to be a reasonable proxy for these. The fatality rates in Iraq were higher for the US military (between 2003 and 2007: from 5.8 to 7.2 per 1,000 for US troops, and 1.9–8.5 per 1,000 for UK troops), but conversely in Afghanistan the rates were higher for UK forces (between 2006 and 2009: 4.8–6.3 per 1,000 for US troops, 5.8–10.8 per 1,000 for UK troops). Thus since 2006 fatality rates have been similar (Rona, Jones, Fear, et al., 2012).

#### Structural and setting characteristics of UK and US military

US and UK forces deployed are demographically different from each other. US forces tend to be younger, of lower rank, and contain more reservists, who are believed to have increased vulnerability to post-deployment mental health problems in both countries. The US forces have a lower leader to enlisted soldier ratio, which may be a meaningful factor as good leadership appears to be protective of mental health. For the Iraq 2003 main cohort study, it was shown that more than two thirds of the UK service personnel had been on previous deployments (Hotopf et al., 2006) in a range of settings, whilst the equivalent US study's sample showed fewer than 10% had had previous deployment experience (Kilgore, Stetz, Castro, & Hoge, 2006). Thus, the UK personnel had more experience of the stresses of military deployments, and so might have been more resilient to these stresses.

As will be discussed later, when Harmony Guidelines on cumulative deployment length are broken, UK forces are more likely to develop PTSD, even after accounting for extra combat exposure. The average UK tour of duty is 6 months, whilst the average US tour lasts 1 year. The US Mental Health Advisory Team has found a linear increase in the relationship between number of months deployed and having a mental health problem, with the highest risk times at eight, nine and 10 months (though there is some decrease in the latter months after this, which may be due to optimism regarding the forthcoming return home, or by psychiatric evacuations in the early months of the deployment). Partly in response to these findings the US tour length has recently been reduced to 9 months (Mental Health Advisory Team [MHAT] 2008). Thus, differing tour lengths may contribute to international differences in Armed Forces mental health.

We can also speculate that easy access to health care might be playing a role in the reporting of symptoms. There is universal health care in the UK, which means that having an injury or disability whether service related or not makes no difference to one's access to free health care (it does however make a difference to one's entitlement to a war pension). In contrast, in other countries having a service-related disability may make a considerable difference to one's health care entitlement; for example in the US for many service leavers automatic health care is only received for 5 years (recently extended from 2 years). Furthermore, thoughts of future disability claims may possibly be contributing to symptom over-reporting, since a number of US studies have found that many treatment-seeking veterans (53%), especially those seeking disability payments, show evidence of symptom exaggeration on psychological tests and forensic interviews (Freeman, Powell, & Kimbrell, 2007; Frueh, Grubaugh, Elhai, & Buckley, 2007; Frueh & Hamner, 2000) although both the evidence and implications of these findings are hotly disputed (Marx et al., 2012).

#### Cultural perspectives

It is obvious that traumatic events are not confined to the battlefield, and that military personnel may enter the forces having already experienced traumas, or that other traumatic events unrelated to combat may occur during their period of military service. When the rates of PTSD in US and UK personnel who had not deployed to Iraq at the time of study were compared, this was found to be modestly higher (5%) in US personnel, compared to in UK personnel (3.7%) (Hotopf et al., 2006). This was despite the UK personnel being older and having had more experience of deployment than their US counterparts, which one might expect to mean that they had already had more exposure to traumatogenic events. This difference could be due to organisational/political differences that might affect reporting of symptoms, a suggestion perhaps supported by research showing that baseline rates in civilian samples in the UK are lower than those in the US (Kessler & Wang, 2008). Alternatively, it may be that the differences in prevalence rates between countries is due to cultural differences in questionnaire completion; as reported for other psychiatric tests, such as Hare's psychopathy checklist (Cooke, Michie, Hart, & Clark, 2005) and the GHQ (Goldberg, Oldehinkel, & Ormel, 1998). A study is currently under way at KCMHR assessing if the same phenomenon is found for PTSD scoring.

#### Methodological approaches

Methodological factors such as anonymity may also have an impact on the different rates of PTSD reported for different nations (Fear, Seddon, Jones, Greenberg, & Wessely, 2012). British studies are confidential but not anonymous, in order to enable records to be linked and followed up. By contrast the core US Land Combat Studies of Hoge and colleagues are genuinely anonymous (Kok, Herrell, Thomas, & Hoge, 2012). Sundin et al. performed a meta-analysis of 19 population-based studies of PTSD after deployment to Iraq (Sundin, Fear, Iversen, et al., 2010). This showed that anonymous surveys of line infantry units reported higher levels of PTSD compared to studies that are not anonymous. KCMHR therefore performed a randomised controlled trial to investigate this further, which showed there was a statistically significant effect on the reporting of sub-threshold and probable PTSD (adjusted odds ratio [AOR] 3.18) when using an anonymous compared to identifiable questionnaire (Fear et al., 2012). The researchers believed that this was most likely to be due to an underreporting of symptoms in those who were concerned about the consequences of disclosure.

However, since the overall prevalence of PTSD is low in the British military, even making British surveys completely anonymous would not lead to PTSD rates as high as those found in the US military.

## Common mental health disorders

The prevalence of common mental disorder (as defined by the General Health Questionnaire-12 (GHQ-12) scores of 4 or more) in deployed personnel was 19.6%, compared to 19.9% for non-deployed military personnel in the UK main cohort study phase 2 (Fear et al., 2010), confirming a previous report of the same study at phase 1 that deployment was not associated with psychological distress (Hotopf et al., 2006). A report from the main cohort at phase 1 found that deployed reservists had higher rates of psychological distress (26%), than non-deployed reservists (16%) (Hotopf et al., 2006), a difference that was not shown in the more recent cohort study (18.1% compared to 19.9%) (Fear et al., 2010). The OHMNE studies between 2009 and 2011 showed prevalence rates of 21, 17 and 16% respectively, confirming similar rates in theatre as those found in the main studies (Mulligan et al., 2010; Jones, in press-a, in press-b).

The OHMNE Iraq study and Jones et al. study both found an association between female gender and common mental health disorders (Jones, Rona, Hooper, & Wessely, 2006; Mulligan et al., 2010) though this was not found in OHMNE Afghanistan studies and was borderline non-significant in the main survey (Jones, in press-a, in press-b; Rona, Fear, Hull, & Wessely, 2006).

Though infantry personnel were substantially more likely to be PCL cases (e.g., suffering from PTSD) than other personnel deployed to Afghanistan, they were not more likely to be suffering from common mental health disorders.

All OHMNE studies found a strong association between weaker unit cohesion, low morale, and poorer perceived leadership with common mental health disorder (Mulligan et al., 2010), an association also found in an earlier study (Rona, Hooper, et al., 2009) and the main survey (Sundin, Fear, Hull, et al., 2010). A number of explanations have been proposed for the association between subjectively good cohesion, leadership, and morale, and better mental health including the stress-mitigating effect of feeling both physically and emotionally protected by leaders in the unit and also having trust in peers and friends (Jones et al., 2012). Unit support in terms of leadership, comradeship, and feeling well informed reduced the chance of having psychological distress according to an analysis based on the screening study (Rona, Hooper, et al., 2009).

The burden of common mental health disorders were not equally distributed; in the 2010 Afghanistan in-theatre study, within major base locations up to half of personnel within some sub-units reported clinically significant psychological health problems whereas only 5% of other sub-units’ personnel reported such symptoms (Jones, in press-a). Personnel who reported symptoms suggestive of suffering from psychological distress were more likely to report having experienced boring and repetitive work, not getting enough sleep and not having enough time to relax. This phenomenon was also found in another study in which rewarding aspects of deployment such as “doing the job trained to do” appeared protective, whilst unrewarding aspects of deployment such as “boredom” were factors associated with psychological distress (Sundin, Fear, Hull, et al., 2010). Job demand and job control are two related risk factors that may play a role in the aetiology of psychological distress and other psychiatric disorders (Karasek et al., 1998). A study based on the main cohort phase 1 showed that low job control and high job demand were additive risk factors of psychological distress as well as PTSD (Fear et al., 2009).

It is unsurprising that the rates of common mental disorders are higher than those of PTSD within the UK military personnel, since as their name implies, they are the most widespread of mental health conditions. The English household survey of 1997, discussed in the earlier section, revealed that for adults aged 16–24 the rate of common mental disorders was 17.5%, rising to 18.8% for the 25–34 age bracket (McManus et al., 2009). The same caveats to extrapolating too much from these dissimilar studies, however, applies. The direct comparisons between national service veterans and matched controls found that the national service veterans were less likely to have any mental disorder (Woodhead et al., 2011a), whilst that of post-national service veterans found no significant difference in the rates (Woodhead et al., 2011b). However, a recent study comparing the prevalence of common mental disorder in the military to the general working population found that the prevalence of CMD was approximately double in the military with a prevalence of 18.7% in military males and 9.1% for males in the general population (Goodwin et al., in press). The prevalence was higher in females than in males in all of the samples. It may be that this is due to a true difference, confounding, or selection bias. Reporting bias may also be a significant factor: a meta-analysis comparing occupational versus general population studies of common mental disorders, suggesting that there is a systematic contextual bias leading occupational studies to generally over estimate prevalence rates compared to true population samples in which people are studied irrespective of, and independent from, any knowledge of their occupational status (Goodwin et al., 2013). This contextual effect may be causing a systematic bias whenever participants are aware that they have been recruited specifically, in this case because they have served in the military.

Thus, UK studies of military personnel suggest: that their rates of common mental disorder are comparable to or higher than the UK general population, that military exposures do not seem to influence rates of common mental disorders, and that certain groups of the military are far more likely to suffer from psychological distress rather than PTSD, with a suggestion that general aspects of daily work seem to have a greater impact on common mental disorders than any specific military exposure.

### Alcohol and risk-taking behaviours

Fear et al. examined data from the main cohort study phase 1 and found that excessive alcohol consumption was more common in the UK Armed Forces than in the general population even after taking age and gender differences into account, the odds were especially high in the younger age groups, and also more of a problem for women in the military (Fear et al., 2007).

A study of troops deployed to Iraq followed up a representative sample of 941 UK service personnel; after 3 years, alcohol consumption and binge drinking increased over time but the rise was greatest in those who had thought that they might be killed or who experienced hostility from Iraqi civilians (Hooper et al., 2008). However, the association between deployment and increased alcohol use did not seem to apply to reservists (who also had a far lower baseline rate of alcohol use than regulars), suggesting that returning to a more exclusively civilian environment might confer some protection against alcohol misuse.

Using data derived from the main cohort study showed that 13% of personnel reported alcohol misuse using a WHO Alcohol Use Disorders Identification Test (AUDIT) cut-off of 16 or more (usually defined as hazardous use that is also harmful to health). Deployment to Iraq or Afghanistan was significantly associated with the report of alcohol misuse on return from theatre in regulars. Alcohol misuse was greatest among those who had undertaken a combat role. The US military appears to have different attitudes towards alcohol use, nevertheless similar associations are found between alcohol misuse and combat roles on deployment (Jacobson et al., 2008). Australian researchers have found however that alcohol disorders are significantly lower in their armed forces than for the general community (Mental Health in the Australian Defence Force, 2010). It is possible that, at least in part, the excessive drinking post-tour is related to time elapsing from exiting theatre, as excessive drinking was more common soon after returning from deployment (Hooper et al., 2008).

As well as an increase in drinking being noted in those who have previously been deployed, a 2008 study found that there is a positive association with having increased exposure to traumatic events on tour and subsequent risky driving (Fear et al., 2008). Explanations for this might include a feeling of invulnerability having survived a high-threat tour, or the possibility that individuals with a combat role are more prone to take risks than those with non-combat roles.

### Medically unexplained symptoms and mild traumatic brain injury

Numerous studies over the last hundred or more years have found that military personnel returning from war frequently experience not just well defined physical or psychiatric injury, but also rises in the rates of unexplained medical symptoms. Many wars have been associated with their own post-conflict syndromes: during the Boer War soldiers complained more of general fatigue, rheumatic pains and weakness, in the two World Wars the most prominent symptoms were shortness of breath, rapid heart rate, headaches, dizziness and chest pain. By the end of the century symptoms like fatigue, headache, depression and anxiety were the main complaints (Jones et al., 2002). These symptom groups have resulted in names such as “Soldier's Heart,” “effort syndrome” and “shell shock.” In 2008 Jones et al. published a study of First World War veterans who had been given a war pension for the effects of gas but who did not display any clear evidence of respiratory damage (Jones, Everitt, Ironside, Palmer, & Wessely, 2008). A significant number of these veterans also suffered with psychological problems, and this group was further convinced that the effects of chemical weapons were irreversible, potent and debilitating; though these convictions were not borne out by their recorded good physical health. It appeared that the conviction of having been gassed (whether accurate or not) had long-term deleterious effects on veterans’ perceptions of their own health and well-being (Jones, Palmer, & Wessely, 2007).

Shortly after the end of the 1991 Gulf War, reports started to emerge of clusters of unusual illnesses occurring amongst Gulf War veterans. Therefore a UK cohort study (funded by the US Department of Defence) of more than 8,000 serving and ex-serving personnel was undertaken, comparing the health and well-being of Service personnel who had deployed to the Gulf, those who had deployed to Bosnia in 1992 on a particularly dangerous peace enforcement mission (providing an active control group) and military personnel who had been in the UK Armed Forces in 1991, but not served in either the Gulf or Bosnia (for a passive control group) (Unwin et al., 1999). This found that there was no evidence that veterans of the Bosnia mission had any worse health than the rest of the Armed Forces, but that Gulf veterans were more likely to report each of the fifty symptoms that were asked about. This provided conclusive evidence that something had affected the health of the UK Gulf veterans, though the symptoms themselves were non-specific. However despite this evidence of poorer health amongst Gulf veterans, there has not been any accompanying increase in “hard” outcomes, such as death, cancer, or physical disease (Gray & Kang, 2006). The mortality rate of both US and UK Gulf veterans are monitored on a regular basis and we know that up to 2009 it has not increased compared to non-Gulf veterans, with the exception of suicide and accidental death. A number of different explanations have been offered for this Gulf War effect including: anxiety regarding the genuine threat of chemical weapons, exposure to depleted uranium, use of organophosphate pesticides or nerve agents, multiple vaccinations, or use of Pyridostigmine (“anti-nerve gas”) tablets. The majority of these have been robustly disproven, and controversy and uncertainty remain. A 2008 review article postulated that media reporting was likely to have influenced, and to continue to influence the health of service personnel (Greenberg & Wessely, 2008).

The Gulf War Syndrome controversy highlighted the need for improved health surveillance and research to take place in a timely manner in further conflicts. Using data from the Iraq cohort study, no repetition was demonstrated of the substantial increase in multiple symptoms that was reported after the first Gulf War; overall there was no difference between deployed and non-deployed groups in the prevalence of “fair or poor” general health’, though the proportion with multiple physical symptoms was slightly greater in regulars (odds ratio 1.33) in the deployed group than in those who were not deployed (Hotopf et al., 2006). In the reservist group there was a larger size effect (odds ratio of 2.1) in deployed than not deployed reservists. In the same study, similar findings were found in cases of fatigue, with no difference between deployed or non-deployed regular personnel, but a significant increase in the number of those scoring four or more points on the Chalder's 13 item fatigue scale for deployed rather than non-deployed Reservists.

### Mild traumatic brain injury

Mild traumatic brain injury (mTBI) has emerged as an important concern in the US military, and has become the “signature injury” of the Iraq and Afghanistan conflicts (Jones, Fear, & Wessely, 2007). An overall mTBI prevalence of 15% was found in a large survey of US combat infantry personnel deployed to Iraq (Hoge et al., 2008), whilst the prevalence in injured personnel returning from Iraq or Afghanistan who had been exposed to a blast was 40% (Okie, 2005). In the UK the reported overall mTBI prevalence was 4.4%, and for combat troops 9.5%. Only 0.7% had had a head injury associated with a loss of consciousness. A total of 10% of participants had sustained injury but without symptoms of mTBI (Rona, Jones, Fear, et al., 2012). Thus, the prevalence of mTBI was lower in British personnel than US, even when only infantry personnel were included. An estimate taking into account length of deployment, which used to be 1 year in the US military, would have increase the prevalence of mTBI to 10.2 cases for 100-person-years, still lower than the 15% reported in the US (Rona, Jones, Rona, et al., 2012).

### Violence and self-harm

There has been a growing concern about a possible rise in violent behaviour amongst military personnel returning from deployment to Iraq and Afghanistan. The prevalence of post-deployment violence was nearly 13% in a study based on the main cohort study. Violence was strongly associated with pre-enlistment antisocial behaviour. After controlling for this, and socio-demographics and military factors, violence was still associated with a combat role (AOR 2), having experienced multiple traumatic events on deployment (AOR 3.7), and also with mental health problems such as PTSD (AOR 4.8) and alcohol misuse (AOR 3.1) (MacManus et al., 2012).

The study comparing health outcomes of post-national service veterans with age and sex frequency-matched non-veterans found that there was an association between veteran status in males and reporting more violent behaviours (Woodhead et al., 2011b).

Media reports and political concerns have also been expressed about possible higher rates of self-harm and attempted suicide in military personnel. Using a telephone clinical interview with 821 individuals (both serving and ex-serving) nearly 5.6% disclosed a history of intentional self-harm: 2.8% self-harm and 4.7% attempted suicide (Pinder, Iversen, Kapur, Wessely, & Fear, 2011). It should be noted that the study participants were drawn from the first phase of the KCMHR cohort study, with an over-sampling of those who had reported psychological distress in a previous questionnaire, but the analysis took due account of weight of the initial sample. A breakdown of ex-service personnel versus currently serving personnel, using weighted percentages, revealed a lifetime prevalence of attempted suicide greater in the ex-service personnel (7.6% compared to 3.8%) and those acknowledging self-harm (4.4% compared to 2.4%). Though overall rates of intentional self-harm are comparable amongst the serving military and general population (prevalence of attempted suicide of 4.4% and self-harm of 4.9%), it seems to be higher amongst ex-service personnel. A strong association was seen between intentional self-harm and PTSD, as well as being young, having a shorter term of service, increasing childhood adversity. This has added further support for the efforts currently under way that seek to improve awareness of, and access to, mental health services, especially for veterans.

## Issues of common interest between allied forces

### Tour length and “overstretch”

The UK Armed Forces have developed “Harmony Guidelines” which aim to determine the maximum length of time personnel should spend on deployment. These guidelines vary by service. The upper limit for the Army is that an individual should deploy for no more than 12 months in a 3-year period. It was found that when these guidelines were adhered to (which they are in between 85 and 90% of cases) there was no relationship between length of time deployed and mental health in terms of PTSD, psychological distress and multiple physical symptoms. However alcohol misuse was associated with length of deployment below the upper limit established in the “Harmony Guidelines.” When personnel had deployed for cumulatively longer than 1 year in a 3-year period, there was an increase in the risk of PTSD, psychological distress, and harmful alcohol use, but adjustment for combat exposure explained these associations (Rona et al., 2007). This finding may indicate that the effect of breaking the “Harmony Guidelines” may be partly associated with combat exposure. Those who had longer deployments than they had expected were more likely to report probable PTSD, a finding also reported by US researchers (Hosek, 2011). This finding underscores the importance of good communication in the chain of command and the need to carefully consider the possible consequences of changing operational planning during deployment.

A more recent study has confirmed the lack of association between number of deployments and mental disorders in regular Army personnel (Table 1) (Fear et al., 2010). The lack of association might be partly explained by the selection bias of the “healthy warrior effect,” in which those who were unwell would have less chance of subsequent deployment (Wilson et al., 2009), but the fact that the odds ratios were, albeit non-significantly, below one indicate that the relatively small bias reported by Wilson et al. would not have changed the interpretation of this relationship. It may also be that those who have experienced adverse consequences from deployment are more inclined to leave the Service. Thus those with multiple deployments could represent a more resilient group of individuals.

**Table 1:** Association between probable mental disorders and number of deployments to Iraq or Afghanistan, for currently serving regular Army personnel (N=4,098)

	1 deployment (N=1,767)	2 deployments (N=1,109)	OR (95% CI); *p* value	Adjusted OR (95% CI); *p* value	3 or more deployments (N=411)	OR (95% CI); *p* value	Adjusted OR (95% CI); *p* value
Probable PTSD	66 (4.1%)	37 (3.4%)	0.83 (0.51–1.36); *p*=0.47	0.96 (0.58–1.57); *p*=0.86	11 (2.5%)	0.61 (0.29–1.26); *p*=0.18	0.72 (0.34–1.50); *p*=0.38
Common mental disorders	355 (21.2%)	199 (18.3%)	0.83 (0.66–1.05); *p*=0.12	0.90 (0.71–1.14); *p*=0.39	67 (16.8%)	0.75 (0.54–1.05); *p*=0.091	0.80 (0.57–1.13); *p*=0.20

### Reservists

A further contributor to the differences between PTSD rates in the military may be the differing proportions of reservists in their forces. The nature of reserve service is not always directly comparable between nation's Armed Forces, for example the United States military in addition to the Army Reserve Force also uses the Army National Guard during deployment which normally is restricted to state duties rather than federal ones.

UK studies have found that after deployment reservists are more likely to suffer from probable PTSD and psychological distress than regular forces. It was found that 26.3% of reservists were suffering from common mental disorder in the main cohort study, compared to 19.4% of regulars, and that reservists’ rates of PTSD were higher (6.0%) than in regulars, though still lower than the comparable rate for US forces (Hotopf et al., 2006). Interestingly, reservists report higher levels of combat exposures than regular infantry soldiers. Since reserves embed within regular units it is unlikely that they are actually experiencing more traumas per se, but it may be that reserves have a heightened sense of threat than regulars from the same dangers (Browne et al., 2007).

One potential reason for the less favourable outcomes for UK Reservists might be related to a perception of not being accepted by regulars when deployed with them; but despite this being cited as an issue in studies conducted in the aftermath of the 2003 invasion of Iraq more recent research has found that most reservists report feeling integrated with their regular colleagues (Dandeker et al., 2010). Another explanation that may account for the increased post-deployment distress of reservists than regulars is that when regulars return from a tour of duty, they tend to continue to spend time with those who they have served with, or at least with those who understand and value what they have been doing. In contrast reservist's friends and employers may have little understanding of the reservist's experiences. Social support does seems to be an important protective factor against military stressors and persistent PTSD (Rona, Jones, Sundin, et al., 2012; Schnurr, Lunney, & Sengupta, 2004; Solomon, Mikulincer, & Flum, 1989), and family members and friends with a poor understanding of a reservist's role and experiences on tour may be less likely to give appropriate informal support, with PTSD symptoms being affected by problems at home rather than events on tour (Browne et al., 2007). A UK study found that reservist personnel experienced more difficulties and less marital satisfaction on their return (Harvey et al., 2011), but this may be explained by reservists having more choice than regulars over their deployments, thus those in failing relationships may be more likely to volunteer for a deployment in order to attempt to escape the issues.

Unlike their regular colleagues, reservists may have concerns about their civilian employment whilst on tour. Harvey et al. reported that 40% of reservists who had a civilian job at the time of their call up reported some perceived deployment-related problems with their civilian employment, such as involuntary loss of their job, perceived loss of promotion and responsibility, or lack of support (Harvey et al., 2011). However, difficulties relating to employment were not associated with a significant increase in any of the mental health outcomes examined. In this study, 44% reported feeling poorly supported by the military in the weeks after they returned from deployment, compared with 30% of deployed Regulars. Overall, 60% of Reservists reported some difficulty in post-deployment social functioning in at least one out of three social domains enquired about (military, non-military, and civilian work). Perceived lack of support from the military was associated with increased reporting of probable PTSD and alcohol misuse.

## Interventions and policy

### Prevention

As previously mentioned, the UK Armed Forces Harmony Guidelines limit on the cumulative length of deployment appears to have a protective effect against PTSD, psychological distress and severe alcohol problems (Rona et al., 2007).

The doctrine of the UK military is that the psychological welfare of troops is primarily a chain of command responsibility, to be supplemented by routine medical briefings and when necessary specialist diagnosis, treatment and advice. However other nations rely on healthcare providers to detect mental health problems in troops, sometimes making use of post-deployment mental health screening programmes (Milliken et al., 2007). Part of the rationale behind the UK approach is that it is those who know the soldiers best professionally (their chain of command) who are best placed to notice any changes in their behaviour or distress within the work context, and that many problems can be resolved with practical unit support in the first instance without needing to medicalise normal experiences. The UK Ministry of Defence has postponed a decision on post-deployment screening for mental disorders until a study using a randomised control trial design reports its results regarding screening's effectiveness. Thus a large scale study of post-deployment mental health screening is underway within the UK Armed Forces which was supported by the 2010 Murrison Report (Murrison, 2010).

Many military forces, including the UK but not the US, currently use a system called “third location decompression” (TLD) to help personnel returning from tour mentally and physically unwind with their units, and to help with the transition from tour to home. For the past 5 years, this has taken place at a facility in Cyprus where UK Armed Forces personnel spend approximately 24 hours receiving psychoeducation and taking part in both group and individual activities. It also allows for a controlled reintroduction to alcohol during an evening social function in order to mitigate the potential for post-deployment alcohol misuse. Research looking at the subjective utility of TLD found that whilst 80% of respondents reported being ambivalent or not wanting to go through TLD before decompression; 91% on completion of it reported finding it useful (Jones, Burdett, Wessely, & Greenberg, 2011).

The US military have introduced BATTLEMIND briefing; the aim of this is to give personnel an insight into how their behaviour has altered in order to be most operationally effective, and pointers on how to now modify this behaviour in order to aid a smooth transition back into home life. US military researchers reported that troops who had experienced high levels of combat and received BATTLEMIND reported less distress than those troops who had received a standard post-deployment brief. Therefore the BATTLEMIND was anglicised, and a randomised control trial took place to see whether it helped amongst UK personnel and thus should be rolled out to all British post-deployment troops. Overall the anglicised BATTLEMIND brief was not shown to be effective with UK military personnel and so in the main has not been introduced, however a modest but real beneficial effect of the anglicised version on binge drinking was observed, and thus these aspects of the BATTLEMIND brief have been incorporated into UK post-deployment briefs (Mulligan et al., 2012).

### Detection

Research comparing studies of stigma in US, UK, Australian, New Zealand, and Canadian Armed Forces personnel revealed that the pattern of reported stigma and barriers to care were similar across these nations (Gould et al., 2010). The British in-theatre study conducted in Iraq in 2009 reported that 10% of the deployed force was interested in some additional support, but the majority of these were fearful of asking for it because of the potential detrimental effect on their career and their reputation in front of peers and chain of command (Mulligan et al., 2010).

A number of approaches have been used with British troops in order to lessen the effect of this stigma. These include stress briefing, self-referral and Trauma Risk Management (TRiM). TRiM is a system of post-incident management intended to allow units to detect personnel at risk of developing psychiatric illness following traumatic incidents, and thus give them early support and referral if necessary (Jones, Roberts, & Greenberg, 2003). It is hoped by many in the military that TRiM may also have the secondary effect of reducing stigma, though this has not been formally studied. One of the central tenants of TRiM is that whilst it should be supported by medical personnel, it should be delivered by specially trained unit members of all ranks, in order to ensure that any psychological threats and the risk of developing a mental disorder are addressed and that personnel are adequately supported (Greenberg, Langston, & Jones, 2008). Qualitative work suggests that TRiM appears to be generally acceptable to military personnel, though a cluster randomised controlled trial comparing personnel on six warships using TRiM and six warships not using TRiM, found no significant change in psychological health or stigma scores between personnel in the different ships (Greenberg et al., 2010). It did find that rates of indiscipline were modestly better in those vessels using TRiM. It should be noted however that in the 12–18 months that these personnel were being studied, the vessels only encountered low numbers of potentially traumatic events.

### Treatment

Within the UK Armed Forces personnel experiencing mental health difficulties are assessed and treated at military Departments of Community Mental Health (DCMH) by psychiatrists, psychologists and psychiatric nurses, these can be either uniformed or MoD employed civilian staff. Inpatient care in the UK for military personnel is provided by specific NHS hospitals with contracts with the MoD, with close liaison between military mental health professionals and the NHS. Personnel requiring help whilst on operations abroad are seen by Field Mental Health Teams. When possible personnel are managed in theatre, but on occasions they are medically evacuated from theatre in order to have treatment in the UK.

Military personnel are usually referred to DCMHs by military General Practitioners, though they can also gain access via their chain of command or unit padres. A self-referral study is currently being piloted.

A range of evidence based treatments including Cognitive Behavioural Therapy (CBT), Eye Movement Desensitization and Reprocessing (EMDR) and other specialist treatments are available at the DCMHs, in addition to psychotropic medications when augmentation is necessary. DCMH staff liaise closely with both the individual's chain of command and Unit Medical Officer to ensure occupational health is preserved, whilst maintaining standards of medical confidentiality (Gould, Sharpley, & Greenberg, 2008; Pook, Tarn, Harrison, McAllister, & Greenberg, 2008).

When personnel, for whatever reason, leave the military, their care is taken over by the National Health Service (NHS). Although veterans generally suffer from similar mental health disorders from the rest of the community, evidence suggests that some veterans are reluctant to seek help from civilian health professionals due to concerns that they will lack understanding of military life or the context of their injuries, and many veterans have expressed the desire to work with therapists and doctors who have expertise or experience of service in the military (Iversen & Greenberg, 2009). Therefore a Community Veterans Mental Health Pilot project providing expertise in military mental health care for veterans was recently evaluated by Sheffield University (http://www.sheffield.ac.uk/cpsr/projects/comvets); its findings were supportive and thus the project has been extended. Additionally, a charity called Combat Stress has received extra funds from the MoD in order to support its work providing assessments and specialist short-stay treatment for veterans (http://www.veterans-uk.info/mental_health), following the Murrison report.

Due to research findings that post-deployment mental health issues were particularly high in reserve personnel (Browne et al., 2007; Hotopf et al., 2006) the Reserves Mental Health Programme (RMHP) was launched in 2006. This is open to current or former members of the Reserves who believe that an overseas operational deployment may have adversely affected their mental health. Mental health assessments are offered, and if a combat-related mental health condition is diagnosed then outpatient treatment is offered via the most appropriate DCMH. A recent clinical follow-up study of reserve forces personnel referred from the RMHP found that PTSD symptoms were significantly improved by treatment at these centres (Jones et al., 2011).

## Conclusions

Despite the intense tempo of UK military operations over the past 9 years, mental health of the UK Armed Forces as a whole seems to remain broadly comparable to the UK civilian population. However, there is no guarantee that the current state of affairs will persist over time, with considerable changes happening with the downsizing of the armed forces, and if operational missions change. Continued surveillance of the mental health of the armed forces remains essential.

A key area of concern is to address the high levels of alcohol misuse with the forces; a challenging issue to tackle given the prevailing cultural perspectives in the military. Further analysis and strategies are needed to combat post-deployment risk-taking behaviour, since this is an avoidable area of mortality and morbidity.

More direct international comparisons of military mental health are likely to be helpful since national differences (for example, whether to screen for post-deployment mental illness, and optimal deployment lengths) are difficult to study within one nation due to ethical and practical barriers to conducting such research.

Another fruitful area may be looking at the lessons learnt by the civilian sector which is increasingly becoming involved in conflict zones, for example, in the form of private security contracts (Messenger, Farquharson, Stallworthy, Cawkill, & Greenberg, 2012), media organisations (Browne, Evangeli, & Greenberg, 2012), and nursing staff. Commercial pressures may encourage some of these organisations to view support differently from how the Armed Forces do, which may be a source of both challenge and innovation to current practices.
